# On a Method For Reconstructing Computed Tomography Datasets from an Unstable Source

**DOI:** 10.3390/jimaging6050035

**Published:** 2020-05-19

**Authors:** Nicholas Stull, Josh McCumber, Lawrence D’Aries, Michelle Espy, Cort Gautier, James Hunter

**Affiliations:** 1 Los Alamos National Laboratory, Los Alamos, NM 87545, USA; espy@lanl.gov (M.E.); gautier@lanl.gov (C.G.); jhunter@lanl.gov (J.H.); 2 Phoenix, LLC., Monona, WI 53713, USA; josh.mccumber@phoenixwi.com; 3 US Army CCDC-Armaments Center, Picatinny Arsenal, NJ 07806, USA; lawrence.j.daries.civ@mail.mil

**Keywords:** neutron radiography, computed tomography, image processing

## Abstract

As work continues in neutron computed tomography, at Los Alamos Neutron Science Center (LANSCE) and other locations, source reliability over the long imaging times is an issue of increasing importance. Moreover, given the time commitment involved in a single neutron image, it is impractical to simply discard a scan and restart in the event of beam instability. In order to mitigate the cost and time associated with these options, strategies are presented in the current work to produce a successful reconstruction of computed tomography data from an unstable source. The present work uses a high energy neutron tomography dataset from a simulated munition collected at LANSCE to demonstrate the method, which is general enough to be of use in conjunction with unstable X-ray computed tomography sources as well.

## 1. Introduction

The first recorded X-ray radiograph was published in scientific literature by Wilhelm Röntgen in 1895 [1], a discovery that laid the foundation for the medical field of radiology and earned him the first ever Nobel Prize in Physics in 1901 [2]. The theoretical basis of X-ray computed tomography (CT) dates to no later than 1917, when the first work on the Radon Transform appeared in the literature [3]. In 1972, the first commercial CT machine was developed by Sir Godfrey Hounsfield [4], and in the decades that followed, applications of computed tomography using X-rays have been the subject of intense study, both in the medical and industrial fields. In parallel, neutron radiography was developed in the 1930s [5,6] and has been used to successfully produce images of reasonable quality in the subsequent decades [7,8]. Additionally, parametric studies to understand image formation in neutron radiography have been performed (for fast neutrons, for example, see [9]). Unlike X-ray radiography, which provides information based on electron density, neutron radiography provides unique contrast based on nuclear cross-sections. In particular, neutron radiography appears very promising for imaging through high-Z materials (see [10] for one example in the case of fast neutrons). There are, however, significant challenges inherent to neutron radiography that have hindered its widespread applicability.

The major challenge addressed in the current work is reliability of sources. A typical high energy neutron radiography system involves either a nuclear reactor (as in some of the earliest examples) or a particle accelerator as is the case at the Los Alamos Neutron Science Center, also known as LANSCE (for more information on the user facility at LANSCE, see [11]). This introduces major barriers in terms of cost, as well as ready availability of sources. Moreover, many of the current linear accelerators in which neutron radiography is feasible are older sources, which were not designed to provide the type of stable beam required for computed tomography.

In the case of X-ray CT, the scan time is on the order of a couple of hours, so as long as the machine is available, repeating a scan is of relatively minimal cost. Additionally, software packages exist that allow the user to set a threshold for successful scans, which will interrupt data collection during the time when the beam is unstable. These advantages are not preserved when examining a part using neutron radiography. At the time of this writing, high sensitivity detectors, especially for high energy (>MeV) neutrons, are not readily available. Often the statistics of a typical neutron scan do not allow an experimenter to set a threshold for data collection, which means that every image is taken, regardless of its quality. CT scan time for a large part in a neutron beam can be on the order of days, which greatly complicates rescanning the part. A final point of distinction is that the part is activated by the neutron beam, which is not typical for low-energy X-ray CT (though it has been observed in parts exposed to 6–20 MeV sources).

An additional challenge presented in this work is the difficulty of imaging a low-Z material (such as high explosives, or HE) encased in a high-Z material (such as steel). This challenge is an inherent limitation in X-ray CT, in which beam hardening resulting from the outer material can serve to obscure subtle features of the interior material, particularly if those features exist near the outer extent of the region of interest. Neutron CT does not exhibit this same beam hardening, so while spatial resolution in neutron CT remains a major limitation, the low-Z material can be imaged in its entirety without artifacts obscuring it. Babai et al. in fact demonstrated a capability directly relevant to this work in fast neutron CT using a steel shell with a plastic core in 2014 [10]. The present work illustrates this through examination of a simulated munition, in which a thick steel case is filled with a mock HE charge. This part is challenging to image in X-ray radiography because of beam hardening due to the shell, which obscures features in the HE, particularly on the outer edges of the HE charge. This presents a major obstacle in the interpretation of X-ray CT scans of this part because the examiner will specifically be interested in subtle features in the HE charge, rather than the outer steel shell.

The above motivates the current work, which lays out a set of strategies (in no way exhaustive) to mitigate the consequences of an unreliable source. It is appropriate to note at this time that, while this work will focus on and present data from a neutron radiography experiment at LANSCE, the methods outlined in this work are also widely applicable to X-ray CT in situations where the beam reliability is suspect.

## 2. Results

**Figure 1:**
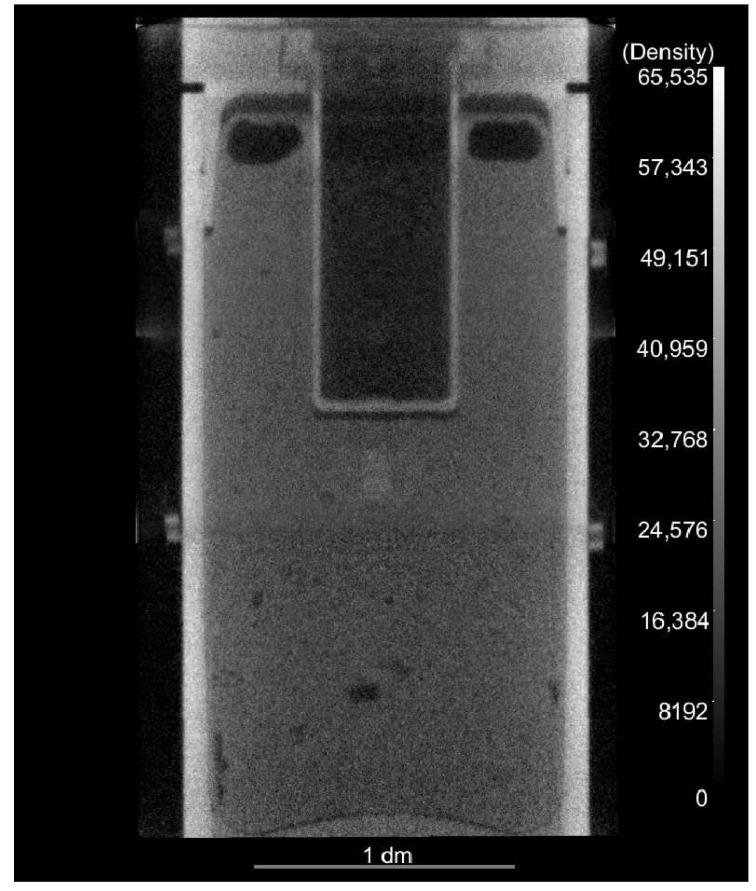
Full side profile of the reconstructed simulated munition, reassembled from 3 separate neutron radiography computed tomography experiments on overlapping segments of the part.

In this work, a high energy neutron radiography CT dataset was collected on a simulated munition. The primary result of this work is a dramatic improvement in the quality of the reconstruction of this dataset. It is worth noting that due to the size of the part (specifically the height), it was imaged in three sessions. All data displayed in the current work, with the exception of the full reconstruction presented in Figure 1, is based upon what will be referred to as the midsection of the scan, or the second session. The midsection CT segment was also used as the test specimen for all pre-processing steps detailed in the next section, with the resulting processing steps being applied directly to the other two segments. Therefore, the results of the lower and upper segments for uncorrected reconstructions (i.e., in which views have been averaged with no further pre-processing carried out) are not presented. However, for completeness, we remark that the level of improvement in reconstruction quality was observed to be similar in other segments.

The final result of the reconstruction, stitched together in Object Research Systems’ Dragonfly (for information on Dragonfly, see [12]), is presented in Figure 1. This side profile image shows numerous voids, one of the primary features of interest when inspecting high explosives (HE), in the simulated HE within the munition. One can also note that the voids become more clearly identifiable and measurable following the image processing steps outlined here. Results displaying this are shown later in the paper. Note that there is a slight discontinuity in grey values between segments, even after interpolation. However, this is indicative of the difficulty in obtaining a seamless interpolation between segments, and is not a reflection of flaws in the processing steps detailed below.

## 3. Discussion

At this time, it is prudent to state explicitly that the current work is limited to industrial applications. In particular, there will be no discussion of medical CT applications, as neutron CT is simply not feasible in such a situation, due to the massive radiation dose associated with exposure to a fast neutron particle beam. In this context, radiation exposure of the part is of secondary concern, while image quality is the primary focus. In the current example of a simulated munition, a typical X-ray CT scan of reasonable quality would require between one and three hours, depending on the source available, while a high energy neutron CT scan of the same part took place over several days in December of 2018. As of the time of this writing, high energy neutron radiography is significantly slower, exhibiting significantly poorer statistics, as compared to its X-ray counterpart, due in large part to the difficulty of detecting fast neutrons. Significant research is ongoing in scintillator development for neutron CT, both in the context of thermal and fast neutrons [13,14,15,16,17].

As previously noted, neutron CT presents a number of distinct challenges that are not typically present in X-ray CT. Specifically, aside from a lack of availability of neutron sources, these sources often suffer from beam instability. Also, the temperature can affect the response of the panel, hence affecting the intensity of radiographs over the course of the imaging session. This is true of X-ray CT as well, but is less of an effect, due to the limited time span of a typical X-ray CT scan (a couple of hours) as compared to that of neutron CT (days). Additionally, radiographs captured during beam dropout, both due to short-term fluctuation of the accelerator output and longer-term maintenance operations, will greatly affect the reconstruction if they are not excluded. These will lead to increased noise in the reconstruction, as well as a reduction of sharpness, particularly near the outer reaches of the part, where limited view counts lead to sparsity in sampling. The key consideration in this work, therefore, is what methods can be used to improve the reconstruction without introducing additional image quality issues, such as blurring and unacceptable noise levels. Additionally, this work and the method outlined herein also motivates some key considerations in experimental design when utilizing neutron radiography.

First and foremost, it is important to ensure that you have captured a full set of radiographs sufficient for a CT reconstruction. This is directly impacted by the instability of the beam. Scheduling in accelerator facilities can be challenging, and staff and facility costs make repeating scans impractical, particularly in high-impact applications. For this reason, the scan should include multiple rotations of the part. This reduces the chance that a scan will have been interrupted in every rotation for a single view by beam instability. However, even if a single view is missing, it can be compensated for (albeit not ideally) by interpolating between adjacent views to insert an artificial view to complete the data set. If multiple views are missing, the scan may need to be redone.

An additional consideration is noise, which is inherent to any imaging system. Typically, the approach used in CT is to take more averages at each view. However, with neutron CT, the integration time for a single image on a digital detector is on the order of 20 s, and the typical scan time for a single object may be several days, which limits the ability to average. However, by taking multiple rotations in a single scan, one can (in essence) treat this as averaging to reduce noise. However, image processing techniques beyond very simple mathematical operations can also increase noise and blurring, which means that any post-processing steps should be kept to a minimum, beyond simple arithmetic operations. The approach taken in this work aims to strike a balance between blurring (due to, among other things, ring removal), noise (inherent to the system), and improvement in image quality of the reconstruction presented.

## 4. Materials and Methods

The current work presents the results of a neutron CT experiment carried out at the Los Alamos Neutron Science Center (LANSCE), on Flight Path 60R (for information on the neutron spallation source in Flight Path 60R, see [18]), and results of processing steps used to improve the quality of the reconstruction. To illustrate this, example data from a neutron radiography CT is presented from images of a simulated munition. The simulated munition, provided by the US Army CCDC-AC and Phoenix, LLC, is of height 16.125″, outer radius 6.0625″, and steel wall thickness of 0.3125″. The simulated munition was filled with mock high explosives (HE). The goal of the work was to achieve adequate contrast and resolution to resolve inclusions and or voids in the mock HE. The simulated munition was imaged using neutrons of average energy 3 MeV (with an energy range of 0–800 MeV) at LANSCE FP-60R. It would be challenging to scan with a standard industrial X-ray source of energy less than 450 keV. This data was collected over a period of several days in December of 2018, in 3 segments (due to the part height and limited beam profile). The panel used was a Perkin-Elmer 1621 panel, with a pixel pitch of 200 microns, with an attached 2.5 mm thick PP + ZnS:Cu scintillator from RC Tritec (for additional details, refer to [8]). The scintillator was thick, a design choice made to improve detection efficiency, but at the expense of spatial resolution.

The data pre-processing was carried out using Matlab [19], with additional processing steps carried out using Recon, a LANL-developed and LANL-maintained software package specifically developed for reconstruction of CT data sets (for more information, please contact the corresponding author). To understand the fundamentals of the methodology of Recon, the reader is referred to the classical text of Kak and Slaney [20], and also to the text of Buzug [21]. The particular implementation utilized for this work is based on filtered back projection (FBP), using the special case of a parallel beam geometric configuration. The subsequent discussion of methods/materials will incorporate simple experimental design considerations, along with motivation for the pre-processing steps taken.

In acquiring data on a source, which may vary over time, it is important to design the experiment to maximize the chances of having a complete data set. In the context of CT, the most obvious remedy is simply to take redundant scans, by rotating the part multiple times. It is reasonable to assume that beam dropout is either randomly distributed in time (for intermittent dropouts) or present over extended periods (as in regular maintenance operations), which means redundant scans is a reasonable strategy for ensuring you collect a complete set of views.

The neutron source was approximately 21 m from the part, and the panel was approximately 10 cm behind the part. The neutron beam was effectively collimated to 3 cm in the vertical direction and 7 cm in the horizontal direction, resulting in an L/D ratio of approximately 700 vertical and 300 horizontal [8]. Thus, we are able to use a parallel beam approximation in the reconstruction. In the experimental data presented in the subsequent section, the design called for a total of 4 rotations of 360° with 1080 views each. However, the entire data run of 4320 views was collected in only one case, while others were truncated after 3884 and 3419 views (i.e., a bit over 3 rotations). Each image was captured over a 20 s integration time to allow for sufficient image quality (statistics).

**Figure 2:**
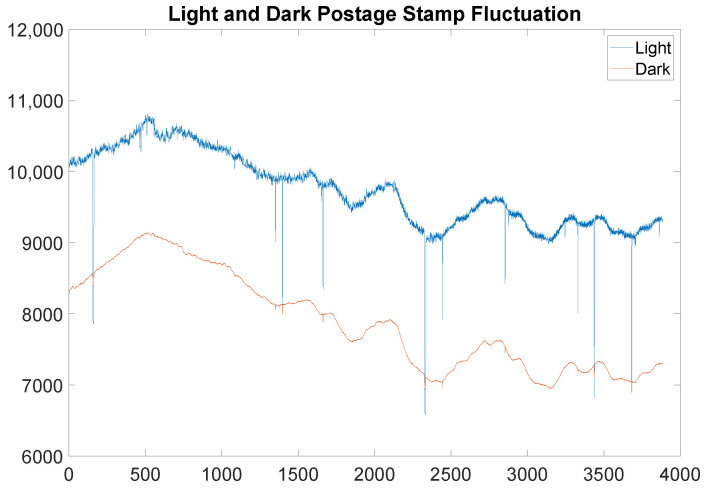
The above graph shows real light and dark postage stamp fluctuation over the time scale of a scan of the middle portion of the simulated munition. The light postage stamp has higher counts throughout, except when the beam drops out entirely. Note the long-term fluctuation is on the order of 15–20%.

The first step in reconstructing the data is to choose a light and a dark region (postage stamp) from the radiographs. It is important the dark postage stamp be chosen from an area that is never illuminated by beam on the panel, while the light postage stamp is chosen to be in open beam throughout the part’s entire rotation. A statistical measure of the data in the postage stamps, in this case the median, is then extracted, as shown in Figure 2. In the case of a short X-ray CT scan, these postage stamps will have relatively flat statistics throughout the scan. The same is not true for neutron radiography CT scans, which may last days, as depicted in Figure 2. Temperature fluctuations on the panel and beam drops are also obvious from these statistical measures of the data.

**Figure 3:**
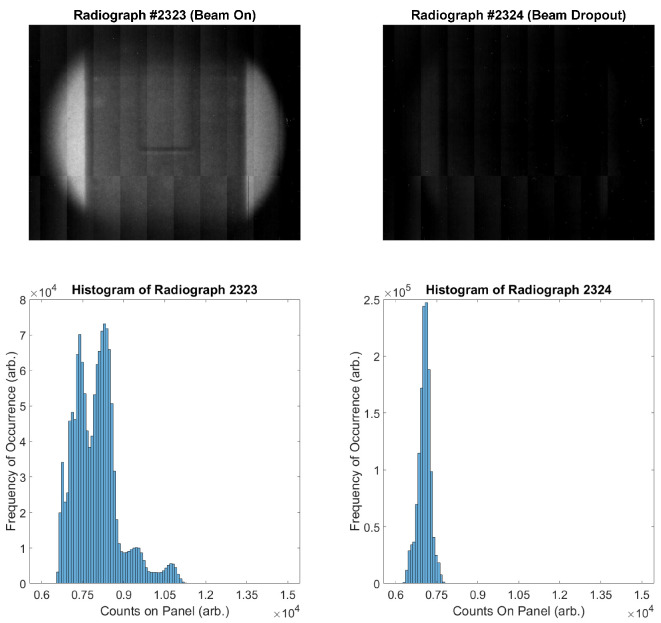
Consecutive neutron radiographs taken during the experiment. **Top Left**: Radiograph #2323, in which the beam is performing as expected. **Top Right**: Radiograph #2324, in which nearly total beam dropout has occurred. **Bottom Left**: Histogram of Radiograph #2323, restricted to the range of [6000, 15,000] for clarity. **Bottom Right**: Histogram of Radiograph #2324, restricted to the range of [6000, 15,000] for clarity.

In fact, it is even clear from Figure 2 that there are images in which the beam is actually off throughout the entire 20 s image. An example of a reasonable image, juxtaposed against an image captured during beam dropout, is presented in Figure 3. It is worth noting that these are consecutive images captured approximately 20 s apart. Additionally, while it may be suspected that there is information that can be retrieved in an image in which beam dropout has occurred, the histograms presented in Figure 3 show that very little information is preserved when beam dropout occurs. Notice that while the range has been restricted to [6000, 15,000] for clarity in both the images presented and the histograms, there were very few responsive pixels in either image registering outside of this range during this scan.

Because of the continuous downward fluctuations, setting a threshold to detect beam dropouts is not possible. For example, referring specifically to the plots of Figure 2, setting an acceptance threshold of 9000 counts will include small dropouts, while setting the acceptance threshold of 10,000 counts would omit real data. Hence, the experimental design must include redundancy of images specifically to compensate for this effect under current experimental constraints.

Pre-processing begins by determining which scans are acceptable and which cannot be used for CT reconstruction. This can be accomplished in a number of ways. Manually tabulating the scans in which beam dropout is observed is both time-intensive and costly for large experiments, and so a more automated and streamlined approach was adopted. The parameters and resulting approach are described below.

The first parameter determines the window length of a median filter that is applied to the median of the data in the light postage stamp. This serves to roughly detrend the data, as well as smooth it. The standard deviation σ of the detrended data is then computed as a constant upon which to base additional parameters. The second parameter, which we will call $T_{rel}$, provides a tolerance of variation between subsequent scans. The third parameter, which we will call $T_{abs}$, provides a tolerance of variation from 0 that the detrended scan data can be. The fourth parameter, which we will call $T_{perc}$, is a decimal value constraining the deviation allowed between the last accepted scan and the current scan.

To be more precise, let $a_{n}$ be the median value of the data in the light postage stamp in view *n*, and let $a_{\ell }$ denote the corresponding value for the last accepted view. Additionally, let $a_{n}^{\prime }$ and $a_{\ell }^{\prime }$ denote the median value of the data in the light postage stamp as above, after applying the median filter. There are, then, a number of conditions that are used to determine whether a scan is acceptable. In particular, we have the following:

**Remark 1.**  *A scan is accepted for further analysis if both of the following conditions (1) and (2) are met:*

*1.*  *All of the following constraints are satisfied:*
  *(a)*  *n is no greater than the number of views taken in the dataset;*
  *(b)*  *The median value of the data in the light postage stamp is strictly greater than that of the dark postage stamp;*
  *(c)*  *And either of the following are satisfied:*
    *i.*  *$|a_{n}^{\prime }-a_{\ell }^{\prime }|<T_{rel}\sigma$;*
    *ii.*  $|a_{n}^{\prime }|<T_{abs}\sigma$
*2.*  *$|a_{n}-a_{\ell }|<T_{perc}a_{\ell }$.*

The above Remark is the foundation of the pre-processing detailed here. The first condition 1(a) requires a bit of explanation. It might appear a trivial statement. However, this condition is specifically chosen to stop the analysis after the actual number of views acquired, which may differ from the total number of views planned in the experimental set up or indicated by values recorded in the technique sheet.

**Figure 4:**
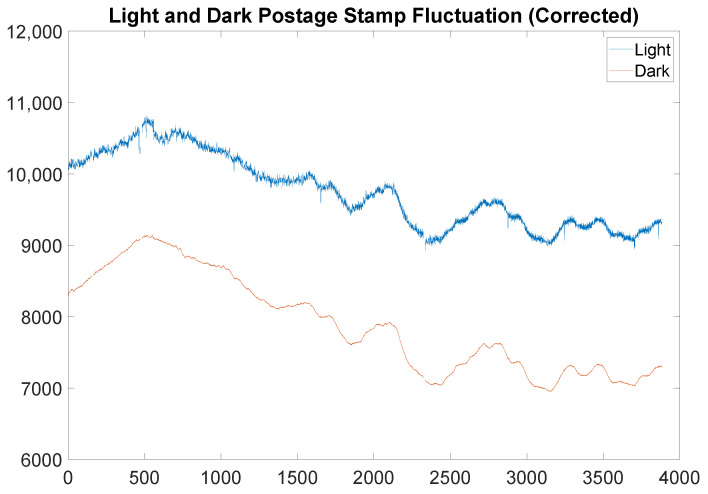
The above graph shows real light and dark postage stamp fluctuation over the time scale of a scan of the middle portion of the simulated munition after applying the filter prescribed in the Remark. For reference, the parameters used were a window length of 21, $T_{rel}=2$, $T_{abs}=2$, $T_{perc}=0.03125$.

While it does not exclude every scan with beam fluctuation (which is functionally impossible), it excludes all radiographs with beam dropout, and in fact is perhaps too aggressive, depending on the application. It is noted that this may in rare circumstances remove some high dose projections in the beam, due to the absolute values in conditions 1(c) i and ii, as well as condition 2. However, this was not encountered in the current experiment. Moreover, unless the parameters $T_{rel}$, $T_{abs}$, $T_{perc}$ are set very aggressively, this is not anticipated to occur. If, in a specific application, this effect is observed, then the absolute values may be omitted as needed to suit the experimental data (though minor modifications would be required, such as condition 1(c) ii. being replaced with the condition $a_{n}^{\prime }>-T_{abs}\sigma$). The key takeaway from the Remark is that a scan is acceptable as long as it is not too dramatically different from the previous scan or the previous accepted scan and that it is not an outlier in the data. The result of applying the filter indicated in the Remark to the data in Figure 2 is presented in Figure 4.

**Figure 5:**
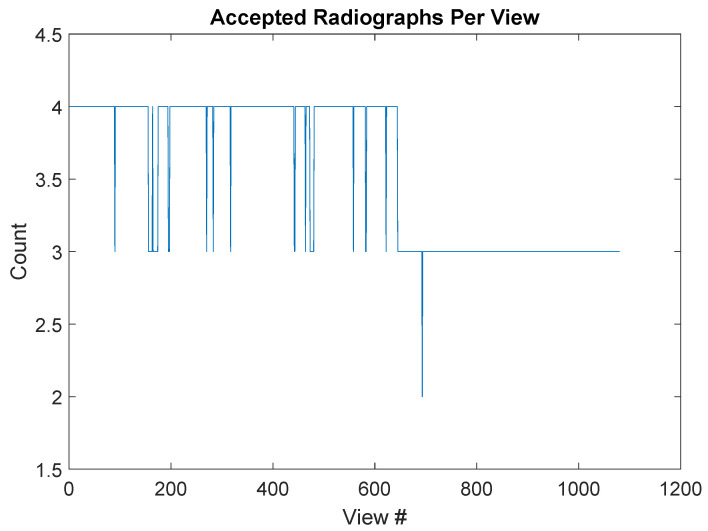
The number of radiographs per view is presented above. Note that even after filtering results for beam dropout and large beam fluctuation, the full set of 1080 views is adequately represented.

The next step is to collapse the dataset down to a total of 1080 views. To do this, it is first important to examine the data and determine whether a complete set of views exists in the accepted radiographs. The plot of accepted radiograph counts against view number is presented in Figure 5. In particular, this graph shows that each view is represented by a minimum of 2 radiographs, with a typical view represented by 3 or more radiographs. It is worth noting that this was not the case in other segments, but in those cases, each view was represented by at least one radiograph. Once we have established that all views are represented by at least one radiograph, we collapse the dataset down to 1080 projections by averaging over all available representations of the same projection. For instance, to form projection 0000, we would average all available views selected from {0000,1080,2160,and3240}.

Assuming this pre-processing is sufficient, the reconstruction process was then started using Recon. The first step is to calibrate the images and convert the calibrated images to attenuation maps using light and dark files. During this step, a bad pixel map is created from the dark and flat-field images, which identifies persistent non-responsive pixels in the panel. These pixels are then replaced with the median value of a 5×5 punctured neighborhood about the non-responsive pixel. The view-by-view history of a horizontal slice of the radiograph (also known as a sinogram) is then computed for each horizontal slice of the radiograph. We then begin the actual reconstruction using a parallel beam algorithm, which in this case was simply filtered back projection.

**Figure 6:**
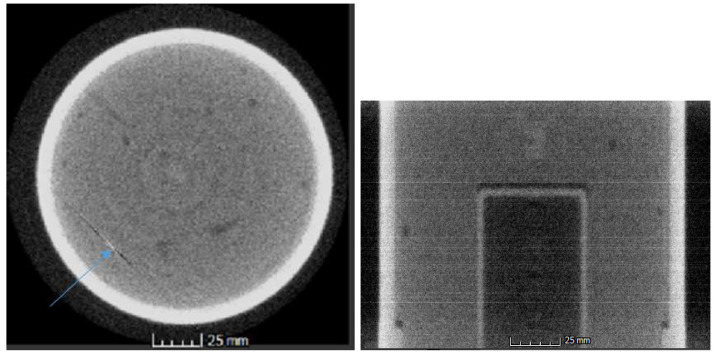
**Left**: A representative slice, exhibiting a hard hit from a neutron in the lower left (indicated by the blue arrow); **Right**: A side profile of the 3-dimensional reconstructed volume. Note the intensity streaks in the volume, which are partially due to hard hits and partially due to beam fluctuation. Visualizations created in Volume Graphics’ VGStudio Max (for information on VGStudio Max, see [22]).

Additionally, we used a three-phase ring removal method. First, a simple median filter with a window length of 31 pixels was applied. Second, a 3-point 10-pass adaptive FIR filter was applied with a cutoff frequency of 0.25. Finally, a frequency based high order Hanning low-pass filter was applied to the noisy row average of the sinogram, with a cutoff frequency of 0.5. In the current context, the frequency is normalized in the interval [0, 1] The result of this preliminary reconstruction is shown in Figure 6.

As can be seen in Figure 6 (Right), the reconstruction exhibits two undesirable traits. First, and perhaps most importantly, there are bright horizontal line segments in isolated layers, which are caused by either an intermittent bad pixel (i.e., a dying pixel), a hard hit from a neutron on the panel, or beam fluctuation. This causes undesirable artifacts (streaks) in the reconstruction slices, as seen in Figure 6 (Left), which in turn degrades the reconstruction quality dramatically. Before proceeding further, this must be dealt with. Note that while Recon will automatically rectify pixels identified in the bad pixel map, it will not, without further user direction, resolve intermittent bad pixels that may only appear in one or two radiographs (which is a key indicator of a hard neutron hit).

The first issue (namely, hard neutron hits and intermittent pixel failures) can be resolved in the reconstruction. By simply replacing outliers (specifically, high outliers) with the median value of their respective neighborhoods, one can filter these hard hits without degrading image quality (these hard hits account for a few hundred pixels in the full 1080-view neutron CT dataset).

The second issue (namely, beam fluctuation) is usually insignificant enough that it can be handled automatically by the Recon software suite. However, if the beam intensity fluctuates by more than a few percent over the course of a scan, this approach becomes less reliable. As a result, the sinograms (and hence the reconstructions) exhibit a striping that is strongly correlated with the fluctuations in beam intensity, as shown previously in Figure 6. To correct this, there are two steps, which are taken in turn.

First, we compute the median of the counts in the light postage stamp of the light file, and the median of the dark postage stamp of the dark file. It is critical that these postage stamps are exactly those chosen for the previous pre-processing steps (i.e., those represented in Figure 4). The data files are then normalized so that the median intensity in the light postage stamp is a prescribed value (in practice, the median intensity of the light postage stamp across the entire set of radiograph views) for each accepted radiograph view. The light and dark files are also averaged and normalized so that the median intensity in the light and dark postage stamps are constant (the median intensity of the light and dark postage stamps across the entire set of radiograph views, respectively).

**Figure 7:**
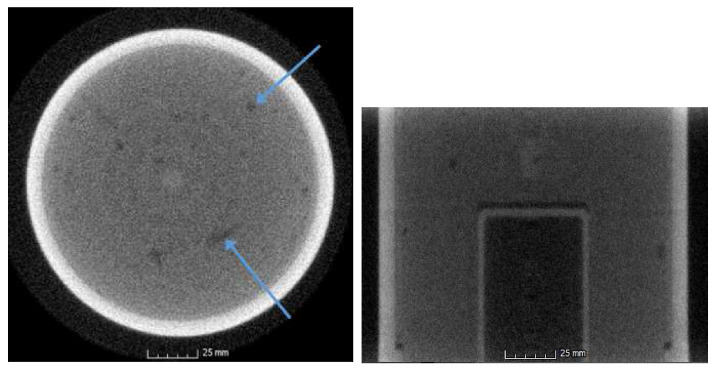
**Left**: No hard hits are observed in any slices in the corrected reconstruction. A sample slice is shown, with blue arrows indicating features examined via lineout (see Figure 8); **Right**: A side profile of the 3-dimensional reconstructed volume. Note that the intensity fluctuations (striping) in the side profile are no longer apparent. Visualizations created in Volume Graphics’ VGStudio Max (for information on VGStudio Max, see [22]).

The set of radiograph views are then written out, as well as the newly normalized light and dark calibration files, and the reconstruction software is used to remove outliers and calibrate the CT views. In some limited circumstances, there may be some residual, albeit very slight, striping in the sinograms (typically on the order of <1%) after the pre-processing steps detailed above. These are due to extremely small residual fluctuations in the projection intensity, in part resulting from the normalization in 64-bit precision in Matlab, with files being written out ultimately as 16-bit raw files. It is worthwhile to note that if the previous steps have been carried out, small fluctuations in sinogram row intensity will be handled adequately by the FBP reconstruction algorithm, and no further sinogram processing should be required. The resulting reconstruction is presented in Figure 7.

**Figure 8:**
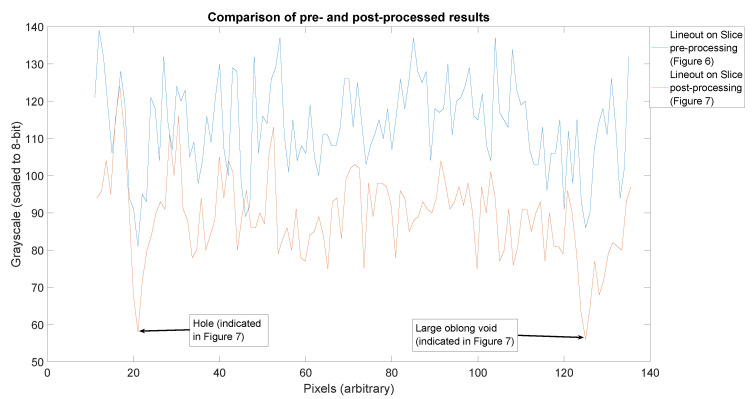
Lineout comparison done between the slice featuring a hard hit (Figure 6) and the slice post-processing (Figure 7).

It is worth pausing for a moment to examine the quality of each of these slices and the relative value of this method. To illustrate this, we take a lineout through the two features indicated by blue arrows in Figure 7, extended in each direction by 10 additional pixels beyond the features of interest. The result is displayed in Figure 8. The grayscale values are slightly different due to the distinctions between processing steps applied. The curve in blue (representing the pre-processed data) is clearly the noisier of the two. Also notable is the degree to which the features of interest are clearly identifiable in the lineouts. These features are indicated by blue arrows. The first feature, a small hole near the outer extent of the slice, is obvious in both slice lineouts. However, when examining the inner void, the noise in the pre-processed slice (Figure 6) obscures the feature, while it is still quite clear in the post-processed slice (Figure 7). This is indicative of the degree of improvement observed in the reconstruction through the methods indicated in this work.

We conclude this section by offering a few remarks on the pre-processing presented in this section. These results are not confined exclusively to neutron CT, but can also be used for long X-ray CT scans, and for CT scans involving time-varying sources. As a final remark, it is worth noting that the reconstruction produced by this routine is of superior quality as compared to the reconstruction produced without taking these pre-processing steps. It is true that the image quality is very slightly degraded by the ring removal step, but the benefit of the pre-processing routine vastly outweighs this small cost. As one can see from the final reconstruction shown in Figure 1, there is a slight transition visible between image segments, but no additional striping artifacts from hard hits or beam fluctuation are visible.

## Abbreviations

The following abbreviations are used in this manuscript:

CT	Computed Tomography
HE	High Explosives
FBP	Filtered Back Projection
